# Supplementary material for: Homeostasis of protein and mRNA concentrations in growing cells
Source: Nat Commun. 2018 Oct 29;9:4496. doi: 10.1038/s41467-018-06714-z (PMC6206055; doi:10.1038/s41467-018-06714-z)
Supplement: Supplementary file 1 — Supplementary Information [file 41467_2018_6714_MOESM1_ESM.pdf]

Homeostasis of protein and mRNA concentrations in growing cells

Lin et al.

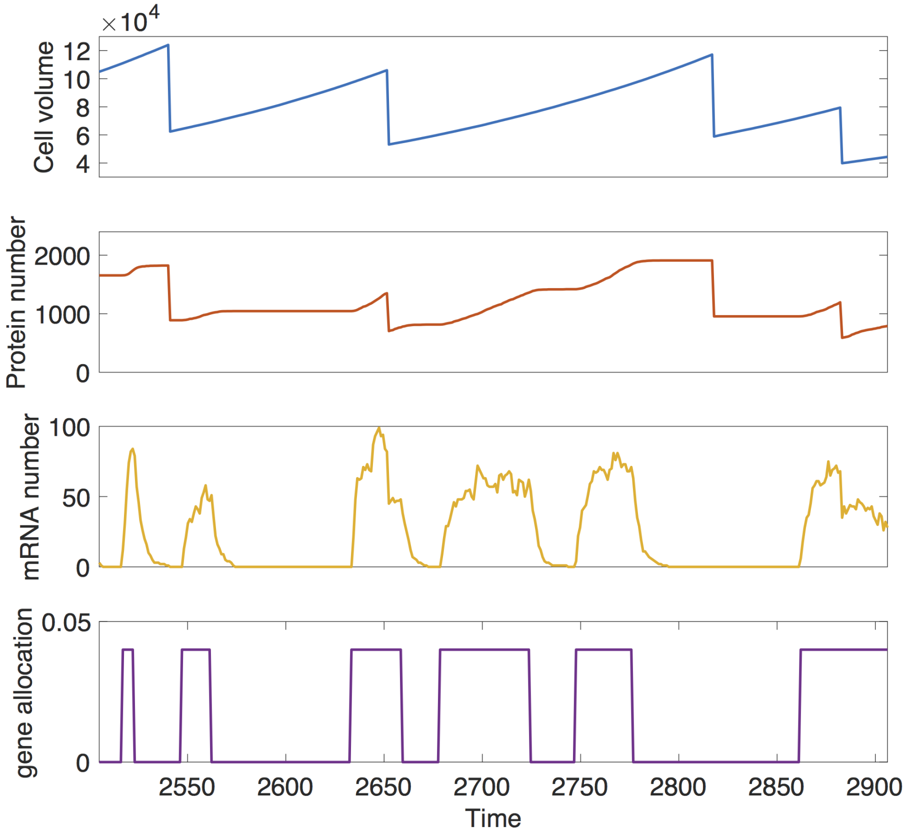

Supplementary Figure 1 The trajectories of cell volume, protein number, mRNA number and gene allocation fraction of one regulated gene. Parameters are the same as Figure 2 in the main text. The time-dependent gene activation rate is set by the concentration of transcription factor $k_g^+ = k_{g0} c_{TF}$. The gene deactivation rate is constant, $k_g^- = 5/T$. T is the generation time. When the regulated gene is active, its gene allocation fraction is 0.04 and the transcription factor's gene allocation fraction is 0.0379. Here we take $k_{g0} = k_g^-/0.0379$.

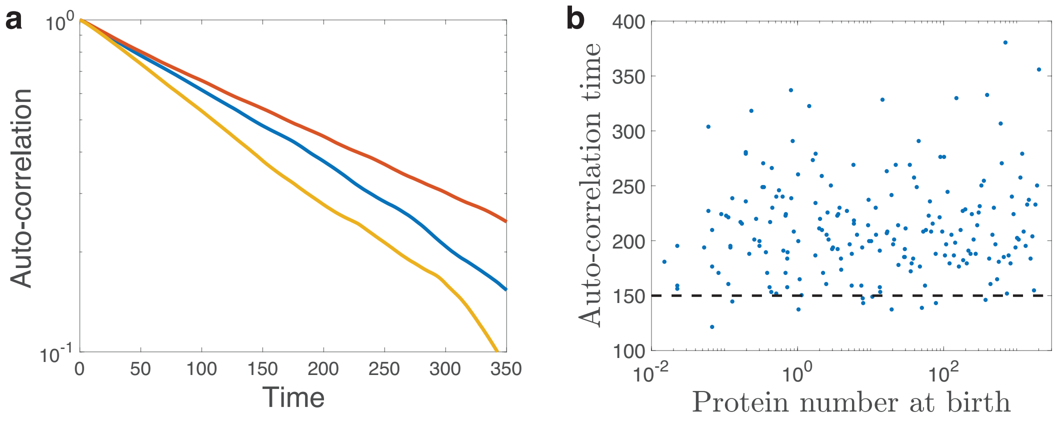

Supplementary Figure 2 (a) We compute the auto-correlation function of protein concentrations, $C(t) = \langle c_i(0)c_i(t)\rangle$. Three examples are shown with the average protein numbers at cell birth are about 1600, 110 and 10.

(b) We calculate the auto-correlation time as $C(\tau_c) = 1/e$. The x axis is the mean protein number at cell birth. We find the auto-correlation time has a lower bound set by the generation time ($T = 150$ min, dashed line).

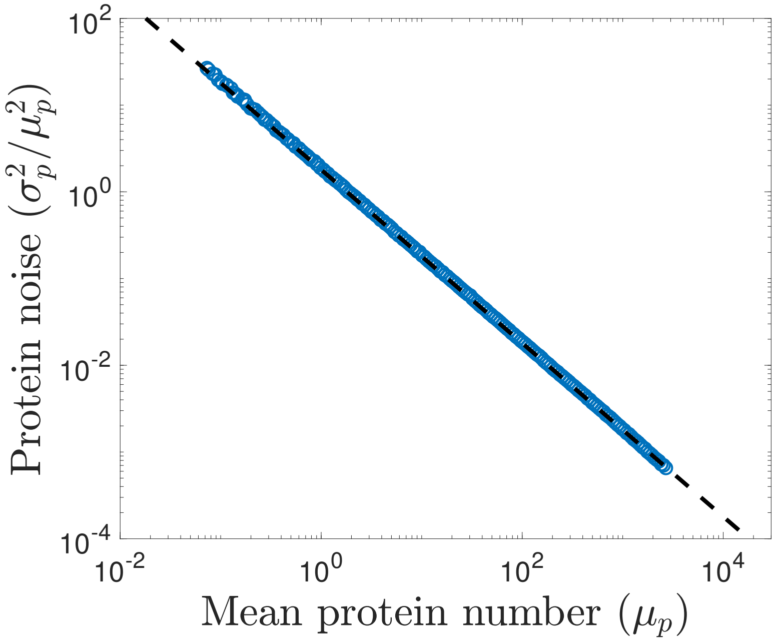

Supplementary Figure 3 We simulate an exponentially growing population based on the model in the section (Models of stochastic gene expression). The noise level is quantified by the squared coefficient of variation of protein concentration, plotted as function of the mean protein number per cell volume. The noise keeps decreasing with a constant slope in the log-log plot, which implies the absence of a global extrinsic noise. The dashed line is the theoretical prediction based on the constant rate model.

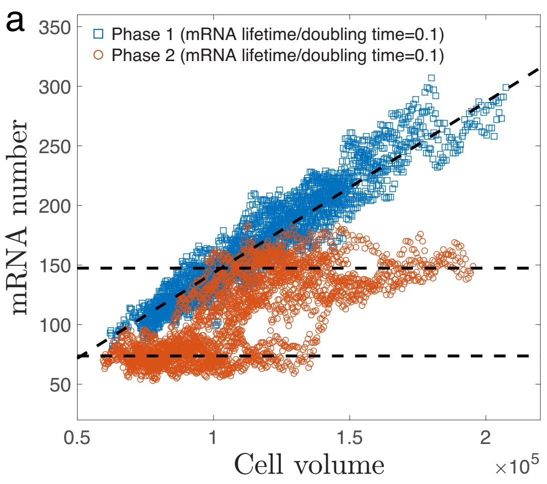

Supplementary Figure 4 We simulate a lineage of growing cells (20 cell cycles), considering a finite duration of DNA replication and sample the mRNA number and cell volume uniformly in time. The doubling time is $T = 150$ min, the C period is 30 min and the D period is 70 min. Other parameters are the same as in the main text. In panel (a), the mRNA lifetime is 10% of the doubling time, while in panel (b) it is 30% of it (these two scenarios correspond to bacteria and mammalian cells respectively). In both cases, we find a linear scaling between mRNA number and cell volume for Phase 1 (RNAP limiting). However, for Phase 2 (gene limiting), we find two plateaus corresponding to gene replication. The dashed lines for Phase 1 correspond to the theoretical predictions of our model (Eq. (5b)). The two dashed lines for Phase 2 are the predictions based on a constant gene copy number before and after gene duplication.

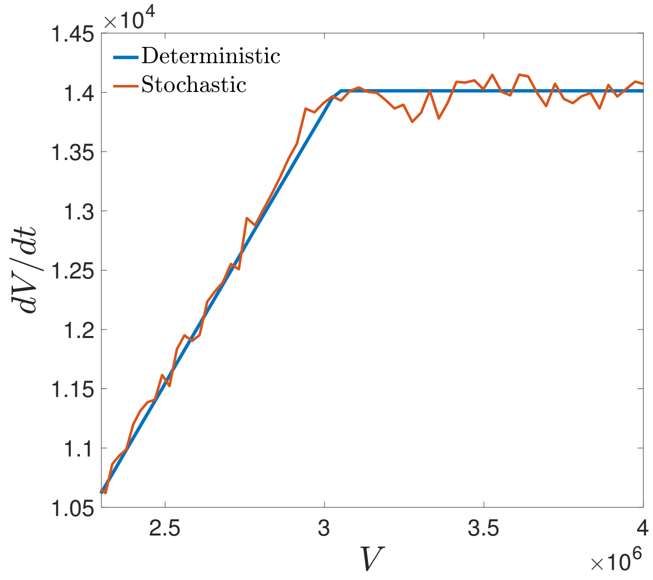

Supplementary Figure 5 We simulate a single growing cell within one cell cycle. We set $n_s = 30$, $r_s = 20$, $\sum_i g_i = 1000$. At $t = 0$, $V(t = 0) = 2.25 \times 10^6$, therefore the protein-to-DNA ratio $\gamma(t = 0) = 2250$. $\gamma_2 = 3.03 \times 10^3$. A transition from exponential growth of cell volume to linear growth is observed as the protein-to-DNA ratio exceeds the threshold value, γ_2. Deterministic trajectories and stochastic simulation are both shown.

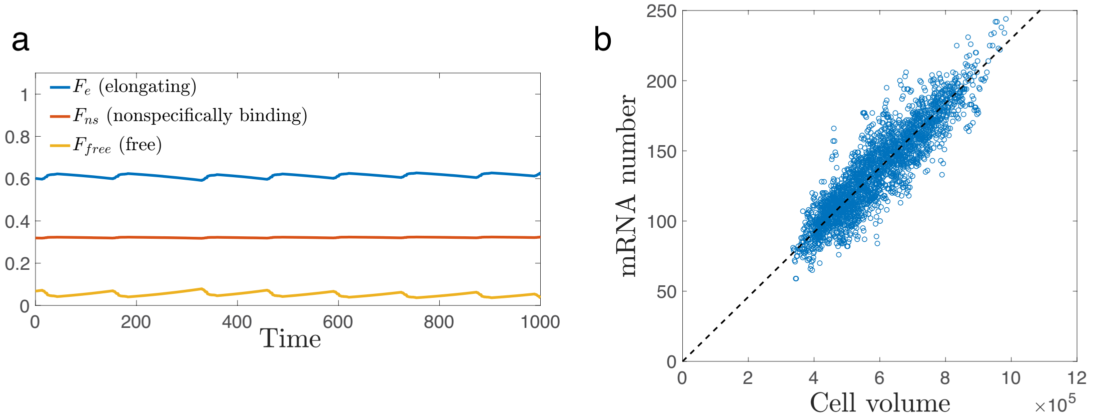

Supplementary Figure 6 We simulate a single lineage of growing cells taking into account the population of nonspecifically bound RNAP. The doubling time is $T = 150$ mins with a C period of 30 mins and D period of 70 mins. To find the fractions of F_{free} at every point in time we numerically solve the self-consistent equation Eq. (31). (a) Trajectories of the fractions of different classes of RNAPs. The small modulation on F_e and F_{ns} (with a coefficient of variation of about 0.01) is due to the small and finite F_{free}. (b) mRNA number is approximately proportional to the cell volume. mRNA number is uniformly sampled in time. $g_{ns} = 1000$ and additional parameters related to the simulation are provided in the Methods.

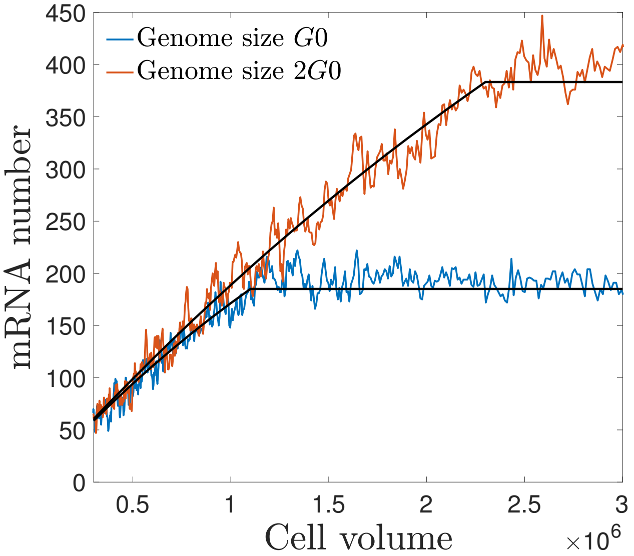

Supplementary Figure 7 We solve the self-consistent equation Eq. (31) to find the fraction of free RNAPs, and the fractions of other classes of RNAPs. The black line is the deterministic dynamics of average mRNA number. The blue and red solid lines are numerical simulations of the full model with stochasticity. Two cells with the same initial volume are simulated and one of the cell has twice a larger genome size. The cells enter Phase 2 (gene limiting) due to steric hindrance. $K_{ns} = 0.08$, $K_s = 0.02$, $g_{ns} = 1000$, $\phi_n = 0.02$, $G_0 = 1000$, $\Lambda = 100$, $n_s = 15$.
